# Environmental Influences on Maternal and Child Health

**DOI:** 10.3390/ijerph14091088

**Published:** 2017-09-20

**Authors:** Katherine P. Theall, Carolyn C. Johnson

**Affiliations:** 1 Department of Global Community Health and Behavioral Sciences, School of Public Health and Tropical Medicine, Tulane University Health Sciences Center, 1440 Canal St., Mailcode 8319, New Orleans, LA 70112, USA; cjohnso5@tulane.edu; 2 Tulane Mary Amelia Douglas-Whited Community Women’s Health Education Center, 1440 Canal St., Mailcode 8319, New Orleans, LA 70112, USA; 3 Tulane Prevention Research Center (PRC), 1440 Canal St., Mailcode 8319, New Orleans, LA 70112, USA; 4 Tulane Center of Excellence in Maternal and Child Health (CEMCH), 1440 Canal St., Mailcode 8319, New Orleans, LA 70112, USA

This Special Issue of *IJERPH* focuses on maternal and child health (MCH), with research that highlights the role of environmental influences on MCH across a range of settings. Importantly, the issue focuses on a broad range of environments, including chemical, natural, built, and social. MCH encompasses health status and well-being of women, infants, children, adolescents, and their families. Their well-being determines the health of the next generation, and can help predict future public health challenges for families, communities, and the healthcare system. However, MCH racial, ethnic, and socioeconomic disparities in morbidity and mortality are significant problems worldwide. These differences are likely the result of many factors which are influenced by a variety of environmental factors, including concentrated poverty which increases exposure to environmental toxins [1], social disorganization and informal social control [2], exposure to violence [3,4], neighborhood cultural context [5], and institutional resources and structural disinvestments [6,7,8]. In many countries, residents of poor communities have access to fewer and lower quality institutional resources that support child development, from safe and affordable food, parks and recreational facilities to child care facilities and schools [9]. Income inequality and income segregation across communities have increased dramatically in most industrialized countries [10], and these developments have elevated concerns among researchers, policymakers, and the public about the impact of concentrated community poverty on the physical and psychosocial development of future generations of children.

Early experiences are biologically and behaviorally embedded, impacting neurodevelopment and health long term [11,12,13,14,15,16], and across generations [17,18,19,20]. There has been an increased focus on such early experiences and their role in shaping health across the life course. In the United States (U.S.), the National Institutes of Health has launched a new seven-year initiative called the Environmental influences on Child Health Outcomes (ECHO) program. ECHO is designed to capitalize on existing participant populations and longitudinal cohorts, taking advantage of the growing number of clinical research networks, technological advances, and data that capture a broad array of environmental factors. Several papers included in this issue focus on environmental toxin exposure among pregnant women and birth and neurodevelopmental outcomes. In a large retrospective U.S.-based cohort study, Mendola and colleagues found that both chronic and acute ozone exposure contributed to an increased risk of stillbirth, with approximately 8000 stillbirths per year in the U.S. attributed to ozone exposure. Polanska and co-authors found, in a large Polish cohort study, a significant impact of environmental tobacco smoke (ETS) during pregnancy on child neurodevelopment in the first two years of life. Other papers in the issue focus on key methodologies utilized in the assessment of environment toxin exposure, such as the intake assessment for lead presented by Fatmi and colleagues or the self-reported oil spill exposure assessment by Harville and co-authors.

Several other papers focus on the importance of the social and physical environments, particularly with respect to childhood obesity prevention and treatment for malnutrition. During the last three decades, the U.S. obesity rate has doubled in adults and tripled in children and adolescents [21]. The widening social inequality gap in childhood obesity in the U.S. suggests a particular vulnerability to environmental factors that increase risk among children who face social disadvantage. Various social and physical environments are considered, including factors in the home, school and community. Godrch and colleagues examine the role of food insecurity on adequate vegetable consumption among rural Australian children, including the specific types of food insecurity—with promotion, location of food outlets, and price playing significant roles in consumption. Complicating the public health picture relative to obesity even more are those countries, such as India, where social gaps are broad, food insecurity (malnutrition) is a way of life within a huge segment of the population, and where one-third of the population is now rated obese. In their commentary, Bazzano, Potts, Bazzano and Mason, discuss the usefulness of ready-to-use therapeutic food (RUTF) which can address the problem of malnutrition in very young children on a community basis with clinical intervention. RUTF has promise worldwide as an aid to combatting this problem. Furthermore, a number of papers examine mechanisms through which the physical and social environments may increase risk. This is important, yet more complex when the focus is on social environmental exposures. While we know that many social environmental factors may play a role in MCH, much remains to be known about the mechanisms through which these factors impact health outcomes. Kepper and colleagues highlight the role of both parent perception of community collective efficacy and neighborhood incivilities in physical activity of youth. Xu and co-authors examine the role of maternal cognition on preschool children’s physical activity and dietary behavior.

The papers within this issue demonstrate the worldwide concern regarding physical social and biological environmental influences on the health of the MCH population, with contributions from various points across the U.S., and from Australia to Pakistan, Japan, Taiwan, and China, and back to European countries, namely Poland, Italy and Austria. The wide range of issues illustrated in this issue confirm the public health seriousness of these environmental influences, as well as the timely publication of this Special Issue on Maternal and Child Health.
